# The past matters: estimating intrinsic hookworm transmission intensity in areas with past mass drug administration to control lymphatic filariasis

**DOI:** 10.1186/s13071-017-2177-6

**Published:** 2017-05-23

**Authors:** Marleen Werkman, James E. Truscott, Jaspreet Toor, James E. Wright, Roy M. Anderson

**Affiliations:** 1 London Centre for Neglected Tropical Disease Research (LCNTDR), Department of Infectious Disease Epidemiology, St. Mary’s Campus, Imperial College London, London, W2 1PG United Kingdom; 2 The DeWorm3 Project, The Natural History Museum of London, London, SW7 5BD United Kingdom

**Keywords:** Soil-transmitted helminths, Lymphatic filariasis, Mass drug administration impact, Interrupting transmission, Transmission models

## Abstract

**Background:**

Current WHO guidelines for soil-transmitted helminth (STH) control focus on mass drug administration (MDA) targeting preschool-aged (pre-SAC) and school-aged children (SAC), with the goal of eliminating STH as a public health problem amongst children. Recently, attention and funding has turned towards the question whether MDA alone can result in the interruption of transmission for STH. The lymphatic filariasis (LF) elimination programme, have been successful in reaching whole communities. There is the possibility of building upon the infrastructure created for these LF-programmes to enhance the control of STH. Using hookworm as an example, we explore what further MDA coverage might be required to induce interruption of transmission for hookworm in the wake of a successful LF programme.

**Results:**

Analyses based on the model of STH transmission and MDA impact predict the effects of previous LF control by MDA over five years, on a defined baseline prevalence of STH in an area with a defined transmission intensity (the basic reproductive number R_0_). If the LF MDA programme achieved a high coverage (70, 70 and 60% for pre-SAC, SAC and adults, respectively) we expect that in communities with a hookworm prevalence of 15%, after 5 years of LF control, the intrinsic R_0_ value in that setting is 2.47. By contrast, if lower LF coverages were achieved (40, 40 and 30% for pre-SAC, SAC and adults, respectively), with the same prevalence of 15% at baseline (after 5 years of LF MDA), the intrinsic hookworm R_0_ value is predicted to be 1.67. The intrinsic R_0_ value has a large effect on the expected successes of follow-up STH programmes post LF MDA. Consequently, the outcomes of identical programmes may differ between these communities.

**Conclusion:**

To design the optimal MDA intervention to eliminate STH infections, it is vital to have information on historical MDA programmes and baseline prevalence to estimate the intrinsic transmission intensity for the defined setting (R_0_). The baseline prevalence alone is not sufficient to inform policy for the control of STH, post cessation of LF MDA, since this will be highly dependent on the intensity and effectiveness of past programmes and the intrinsic transmission intensity of the dominant STH species in any given setting.

## Background

Neglected tropical diseases (NTDs) affect some of the poorest populations in the world [1]. Over the past decade many initiatives have been started to reduce morbidity induced by NTDs in endemic regions [2]. For example, in 2000, the Global Alliance to Eliminate Lymphatic Filariasis (GAELF) was formed, whose aim is to achieve elimination of transmission for lymphatic filariasis (LF) by 2020 [3]. In 2014, 73 countries were identified as needing mass drug administration (MDA) to control LF. To achieve elimination, community-wide programmes providing albendazole in combination with ivermectin or diethylcarbamazine, were started in 62 of these countries. These programmes have been very successful, with 18 of these countries now moving to post-MDA surveillance once the prevalence of LF had dropped to 1% or less [3, 4]. Of the remaining countries, eleven still needed to start their MDA programme in 2014 [4].

LF is frequently co-endemic with soil transmitted helminths (STH) [5]. These intestinal parasites of humans are endemic in sub-Saharan Africa, Asia and parts of South America [6]. They comprise of three main species, i.e. *Ascaris lumbricoides* (round worm), *Trichuris trichuria* (whipworm) and two hookworm species, *Necator americanus* and *Ancylostoma duodenale.* These parasites are transmitted between hosts through the excretion of eggs or larvae into the environment. Re-infection occurs either orally (*Ascaris* and *Trichuris*) or through the skin (hookworm) [7]. Globally, they are estimated to infect 1.45 billion people, resulting in approximately 5 million years lived with disability [8].

Chemotherapeutic treatments exist for all STH species, although with varying efficacies for the drugs of choice [9, 10]. These are good for hookworm and *Ascaris lumbricoides* but less so for *Trichuris trichuria*. Current World Health Organisation (WHO) guidelines regarding STH focus on drug treatment for preschool-aged children (Pre-SAC: 2–4 year olds) and school-aged children (SAC: 5–14 year olds), with the goal of eliminating STH as a public health problem amongst children [10]. Recently, attention and research have started to focus on the broader question of whether MDA programmes alone could potentially break the infection cycle for STH parasites, eventually resulting in parasite elimination [11–14]. Areas where LF elimination programmes are active are likely to have achieved a reduction in both prevalence and intensity of STH through annual community-wide MDA [5, 14, 15] involving the use of albendazole and mebendazole, both of which are active against all three major STH parasites though with varying efficacy [7, 9, 16]. The potential for building upon the existing infrastructure for community-wide MDA programmes in these regions, combined with the projected reduction in STH prevalence and intensity, makes these areas particularly suitable for investigating the possibility of STH transmission interruption through MDA [5, 14, 17]. The current momentum towards achieving NTD elimination provides an opportunity to investigate whether MDA alone can interrupt the transmission of STH.

For the STH species, female worms need to find a male within the host with whom to mate to produce fertile offspring. These exit the host as eggs or larvae and perpetuate the life-cycle. As such, there exists, within the host population, a critical mean adult parasite density for effective sexual reproduction below which the parasite population cannot be sustained [18–21]. The inclusion of this ‘mating function’ within mathematical models of parasite transmission and MDA creates three possible equilibria for the mean worm burden, two are stable (endemic infection and extinction) separated by an unstable state which is defined as a “breakpoint in transmission” [14, 20]. When this breakpoint is crossed, the parasite population moves towards the stable state of extinction in the absence of any immigration of infection into the human population. No further treatment is required once the breakpoint is crossed.

Current WHO treatment guidelines for STH are unlikely to result in the interruption of transmission, as the adult population is not targeted and they create reservoirs of infection [21–24]. This is especially true for hookworm (*Necator americanus*) where a move from targeted strategies to community-wide treatment is imperative to achieve elimination, as the highest prevalence and intensities of infection are typically found in the adult population [11, 12, 25, 26].

The success of interventions aimed at transmission elimination will be highly dependent on the transmission intensity in a given setting, as measured by the basic or intrinsic reproductive number, R_0_. With the use of country- and site-specific cross-sectional treatment coverage and hookworm prevalence data it is possible to use mathematical models of parasite transmission dynamics, to define what level of coverage will result in transmission elimination in defined transmission intensity settings. These models can help inform policy makers on what treatment frequency, coverage levels and programme durations will be necessary to achieve this goal.

The baseline prevalence is highly dependent on the transmission intensity and pattern of parasite aggregation within the human host population, but it is also affected by the past history of MDA treatment programmes. In this paper, we investigate the impact of LF MDA treatment on the prevalence of hookworm infection, and investigate what regimens of MDA coverage in a subsequent elimination study once LF treatment has ceased will be necessary to achieve the interruption of hookworm transmission. It is important to note that LF community-based coverage is rarely recorded accurately so some exploration of a range of options about the past history of MDA coverage is essential in a given location.

## Methods

**Fig. 1:**
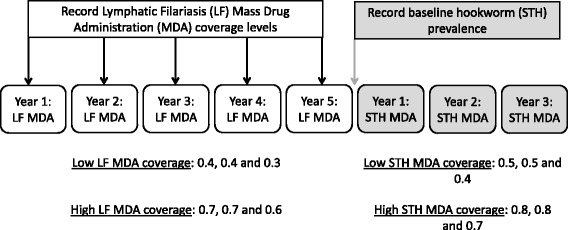
Diagrammatic overview of study design including the data collection times, MDA settings and the simulated interventions

**Fig. 2:**
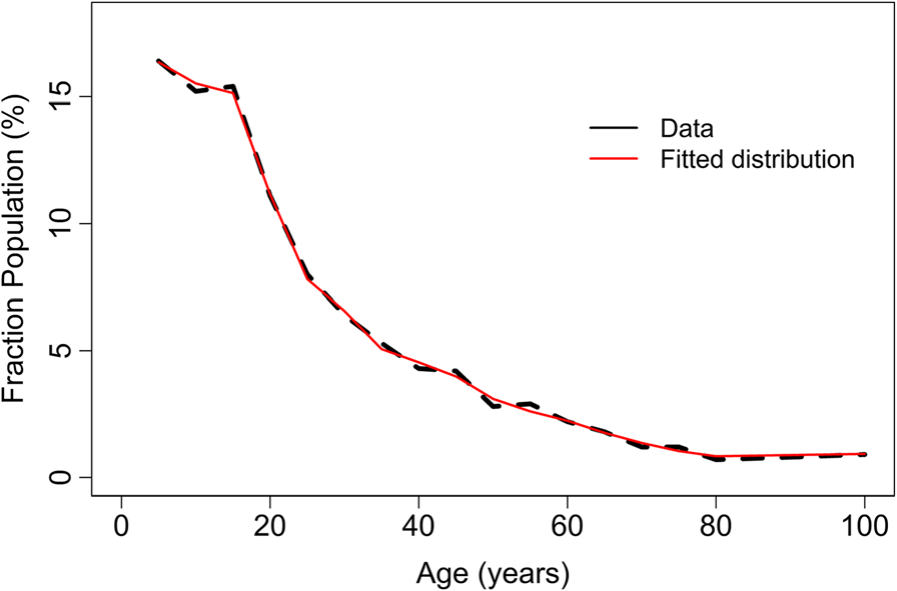
Demography profile of a rural community in Kenya (unpublished data) that was adopted in this study (*black* line shows the data and *red* the fitted distribution)

We assume a scenario where LF programmes have been active for five consecutive years with a follow-up programme of three years to treat hookworm infections. The impact of two LF coverage settings on the prevalence at baseline are explored and the success of four different follow-up STH elimination programmes are investigated (Fig. 1). We have employed a deterministic mathematical model to simulate the LF programmes and STH elimination programme [21, 27]. Recent work has shown that the deterministic age-structured model predictions are in very good agreement with the mean of a stochastic individual-based model [28]. The deterministic model consists of two parts, one describing the evolution of worm burden in individuals within the host population as a function of time and host age, and an equation governing the dynamics of infectious material in the reservoir (eggs or larvae). Infected individuals contribute fertilised eggs into the environment and are re-infected according to the force of infection experienced from the environment. Age structure is essential within the model, as the force of infection experienced by an individual is a function of age and the degree of treatment experienced within the context of MDA is also highly age-dependent. In this study, we included the demography profile of Kenya to represent a typical age-profile of a low- or middle-income country (Fig. 2).

The mean female worm burden for individuals of age *a*, *M*(*a*, *t*), is described as:

$$ \frac{\partial M}{\partial t}+\frac{\partial M}{\partial a}=\varLambda ( a, t)-\sigma M $$

We assume that the sex ratio in hookworm is 1:1, therefore, the total worm burden is 2*M*(*a*, *t*).

The parameter *σ* is the reciprocal of the mean life span of an adult hookworm. The parameter *Λ* defines the force of infection of individuals of age *a* as, and is defined as:

$$ \varLambda ( a)= L( t)\beta ( a) $$

where *β*(*a*) represents the contact rate of a host with age *a* with the environmental reservoir (Table 1). The contribution of infectious material to the environment is also dependent on age, *ρ*(*a*) describes the relative, age-dependent, contribution to the infectious environment, *L* The concentration of infectious environment at time *t* is given by:

**Table 1:** Model parameters of the simulation model

Model parameter description	Value
Aggregation of parasites in hosts	*k* = 0.35 [28]
Relative exposure and contribution to the reservoir (assuming no difference between males and females)	0–15 years old: 0.12; 15–25 years old: 1; 25+ years: 0.07 [28]
Average worm lifespan	2 years (assuming an exponential distribution) [20]
Female worm fecundity	*γ* = 0.02 (assuming exponential saturation) [28]
Survival of infective material in the environmental reservoir	mean = 12 days (exponential survival) [20]
Drug efficacy	0.940 for albendazole [37]

$$ \frac{dL}{dt}=\frac{\psi \lambda}{a}{\displaystyle \underset{a=0}{\overset{\infty }{\int }} M(a)} f\left( M(a), z, k\right)\varphi \left( M(a), z, k\right)\rho (a) d a-{\mu}_2 L $$

The first term is the total flux of infectious material into the environmental reservoir. The parameter *λ* (Table 1) is the mean net output of eggs per female worm in a standard quantity of faeces (e.g. per gram) and the parameter *ψ* represents a conversion factor into the rate of flow into the infectious reservoir. The combined mortality rate of the two infective stages (eggs and larvae) of the parasite in the environment is *μ*_2_. *S*(*a*) is the survival probability of a host from birth up to age *a* and can be expressed in terms of the age-dependent death rate of hosts *μ*(*a*):

$$ S(a)= \exp \left(-{\displaystyle \underset{d=0}{\overset{a}{\int }}\mu \left( a\hbox{'}\right) da\hbox{'}}\right) $$

The age structured model includes MDA treatment with varying coverage levels by age grouping to investigate the impact of regular treatment cycles on the prevalence and intensity of infection. Individuals are divided into four different treatment age groupings; 0–1, 2–4 years (pre-SAC), 5–14 years (SAC) and > 14 years (adults). The coverage of MDA refers to the proportion of individuals treated at random within an age-category per unit of time. Systematic non-compliance issues are not included in this study [29, 30].

For the principle STH species, the mean number of eggs produced by a female worm decreases when the number of worms in a host increases (density-dependent fecundity [31]). The decrease in egg output is highly nonlinear, i.e. decreasing the worm burden by 50%, will not decrease the egg output by 50%. Hence, the mean egg production from a host population with mean worm burden $\overline{M}$ is described as the following function:

$$ f\left(\overline{M}, k, z\right)=\frac{\psi \lambda \overline{M}}{{\left[1+\left(1- z\right)\overline{M}/ k\right]}^{\left( k+1\right)}} $$

where *k* represents the aggregation of the number of worms within a host and is the shape parameter for the negative binomial distribution (Table 1). The parameter *z* is the density-dependent fecundity function where; *z* = *e* ^− *γ*^. Here *γ* determines the severity of the density dependence.

We assume that hookworms, as with most STH species, are polygamous. This means that a single male can reproduce with all females present in a host. For this biological assumption, the mating function is given by May [19]:

$$ \varphi \left( M; z, k\right)=1-{\left[\frac{1+ M\left(1- z\right)/ k}{1+ M\left(2- z\right)/ k}\right]}^{k+1} $$

Finally, the intrinsic transmission intensity, defined by the basic reproductive number R_0_, is defined as the average number of female worms produced by a single worm that reaches the fertile age in a fully susceptible population. R_0_ is defined as in [27]:

$$ {R}_0=\frac{z\lambda \psi}{\mu_2\overline{a}}{\displaystyle \underset{a=0}{\overset{\infty }{\int }}\rho (a)} S(a){\displaystyle \underset{x=0}{\overset{a}{\int }}\beta (x)}{e}^{-\sigma \left( a- x\right)} dxda $$

The prevalence and average intensity of infection of any STH species in the host population is highly dependent on the transmission intensity, R_0_, along with parasite aggregation defined by the negative binomial parameter *k*. However, if there is a history of MDA treatment, the prevalence is expected to be lower than that prevailing in the untreated population, in contrast to the intrinsic R_0_ value, which is independent of prior LF treatment. The higher R_0_, the higher the expected prevalence for a given *k* value.

However, the effective transmission intensity, R_*e*_, is affected by the MDA treatment. We assume that the likelihood of a worm surviving is 1 − *gh*, where *g* represent the proportion of the population treated and *h* represent the efficacy of the drug (i.e. the proportion of worms killed in a host, Table 1) [21].

**Fig. 3:**
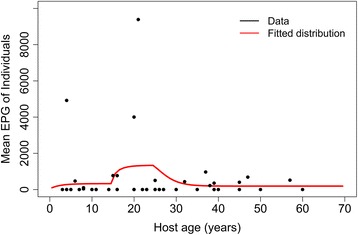
The age-infection profile (*red*) fitted to individual-based intensity data (*black*)

Individual-level intensity data obtained from an epidemiological study of hookworm performed in Tamil Nadu, South India [32] was used to define the shape of the age intensity profile of infection (Fig. 3). The aim of this epidemiological study was to assess the impact of three different MDA strategies (different treatment intensities and frequencies) on the mean eggs per gram (epg) and prevalence and carried out in 45 villages. Details of the study can be found in Sarkar et al. [32]. Stool samples from three different days were collected from all participating individuals and the mean epg and age of the individuals were recorded. The village with the highest prevalence and intensity data was selected to calculate the likelihood function for the age-stratified epg data in order to estimate key parameters such as R_0_ and the rates of infection stratified by age group (Fig. 3) [28]. The village selected for this estimation procedure underwent the most intense MDA treatment.

The parameter R_0_ was varied independently in the analysis to generate the pattern of age related changes in the prevalence of infection observed in different villages. The most likely R_0_ was estimated and then based on this value projections were made of the effects of an STH-intervention programme with defined coverage and duration, after 5 years of LF MDA. It is assumed that nothing changes over this period to influence the intrinsic STH transmission intensity (the value of R_0_) over the entire period of LF and post LF treatment.

Two measures of prevalence are calculated, the true prevalence of female worms in the human host population and the measured prevalence, which has errors due to the limited capabilities of the Kato-Katz method of egg counting and detection in faeces [33]. We assume that the diagnostics are performed using Kato-Katz, which is the most commonly used diagnostic method for STH infection [26], with results based on two independent samples. The measured prevalence takes into account the negative binomial distribution of egg counts per stool sample and the need for the presence of fertilized female worms for the production of viable infective stages.

To explore the intrinsic hookworm transmission intensity prevailing in a locality prior to LF MDA, we parameterize our model with data available from past LF control programmes, including the number of years of prior LF treatment and the coverage level achieved in each year. We also include a measured baseline prevalence of hookworm at the end of the LF programme and at the start of the STH MDA programme, to estimate the intrinsic hookworm transmission intensity (Fig. 1). The past MDA coverage and hookworm prevalence data may be fully cross-sectional by age or simply values for the pre-SAC and SAC age groupings. After the cessation of the STH MDA programme, the model exhibits two types of behaviour; either moving back to the stable endemic state (bounce back) or moving to the transmission and parasite elimination state. These two states are separated by the unstable breakpoint in transmission [14, 20].

## Results

**Fig. 4:**
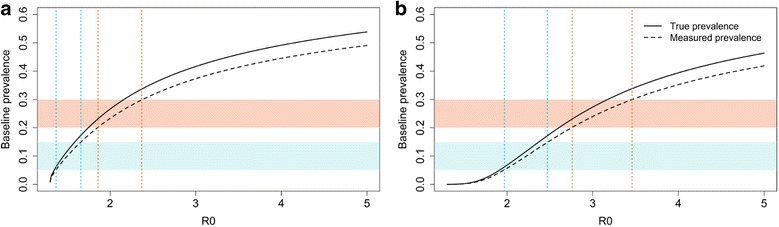
The relationship between R_0_ and the prevalence of infection in pre-SAC and SAC after five rounds of treatment assuming two coverage levels for LF. **a** Assumed LF coverage levels are 0.4, 0.4 and 0.3 for pre-SAC, SAC and adults respectively. **b** Assumed LF coverage levels are 0.7, 0.7 and 0.6 for pre-SAC, SAC and adults respectively. The *dotted vertical lines* indicate the R_0_ regions that match with the measured prevalence regions (*blue*: 5–15% prevalence; *red*: 20–30% prevalence)

The baseline prevalence is affected by the transmission intensity and the coverage of LF treatment as illustrated in Fig. 4, which shows the effects of the value of R_0_, on the baseline prevalence for a defined value of the negative binomial aggregation parameter *k* which was set to 0.35 [28]. Five years of LF treatment prior to STH control are simulated (Fig. 1). For the LF treatment, two different effective coverage levels are included in the analysis: (i) a low LF MDA coverage of 0.4, 0.4 and 0.3 for pre-SAC, SAC and adults, respectively (Fig. 4a); and with (ii) a high LF MDA coverage of 0.7, 0.7 and 0.6 for pre-SAC, SAC and adults, respectively (Fig. 4b). Two measures of prevalence are shown in Fig. 4; true prevalence and measured prevalence which takes into account the negative binomial distribution of egg counts per female worm and the need for the fertilization of the female. To fall within a relatively low measured baseline prevalence range of 5–15%, requires an intrinsic R_0_ value for hookworm of between 1.37–1.67 for low LF MDA coverage (Fig. 4a) and between 1.97–2.47 for high LF MDA coverage (Fig. 4b). A higher baseline measured STH prevalence range was also examined, where a measured prevalence between 20 and 30% at baseline is assumed. To fall within a prevalence range of 20–30%, a higher R_0_ is predicted fall between 1.86–2.37 for low LF MDA coverage (Fig. 4a) and 2.76–3.36 for high LF MDA coverage (Fig. 4b).

**Fig. 5:**
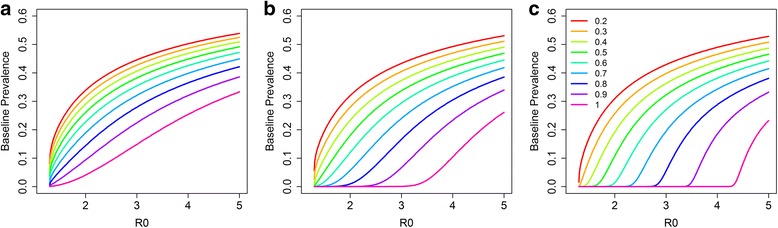
The effects of historic LF coverage on the intrinsic R_0_ values. The colours indicate the coverage levels and different durations of LF programmes that are assumed: two (**a**), five (**b**) and ten years (**c**). Adults are assumed to have 10% lower MDA coverage compared with pre-SAC and SAC)

As illustrated in Fig. 4, the LF MDA coverage has a substantial impact on the hookworm prevalence at baseline. However, the number of treatment rounds are also important to take into account when estimating the transmission setting for STH (the R_0_ value). Figure 5 shows the effects of the duration of MDA programmes (2, 5 and 10 years) and the coverage levels (ranging from 0.2 to 1) on estimates of R_0_ for different baseline prevalences. The effects of coverage levels increase when the programme has been active for five or ten years (Fig. 5a-c). For the lowest investigated duration (Fig. 5a), assuming a prevalence range of 5–15%, the R_0_ is estimated to be between 1.30–1.38, 1.31–1.51 and 1.38–1.79, with a coverage of 0.3, 0.5 and 0.7, respectively. For the longest programme duration, still assuming a baseline prevalence range of 5–15%, R_0_ is expected to be between 1.41–1.63, 1.84–2.13 and 2.52–2.82, with a coverage levels of 0.3, 0.5 and 0.7, respectively (Fig. 5c).

To fall within the baseline STH prevalence of 20–30%, R_0_ is predicted to be higher. For the shortest programme durations, R_0_ is expected fall within the ranges 1.79–2.21, 2.32–2.83 and 3.03–3.60, with a coverage of 0.3, 0.5 and 0.7, respectively (Fig. 5a). The impact of coverage on R_0_ is the highest for the longest programme duration (ten years, Fig. 5c). In this scenario, R_0_ is expected to be 1.79–2.21, 2.32–2.83 and 3.03-3.60, with a coverage of 0.3, 0.5 and 0.7, respectively (Fig. 5c). When the achieved coverage is low, the effects of treatment duration on the estimated transmission intensity are limited (Fig. 5a-c).

**Fig. 6:**
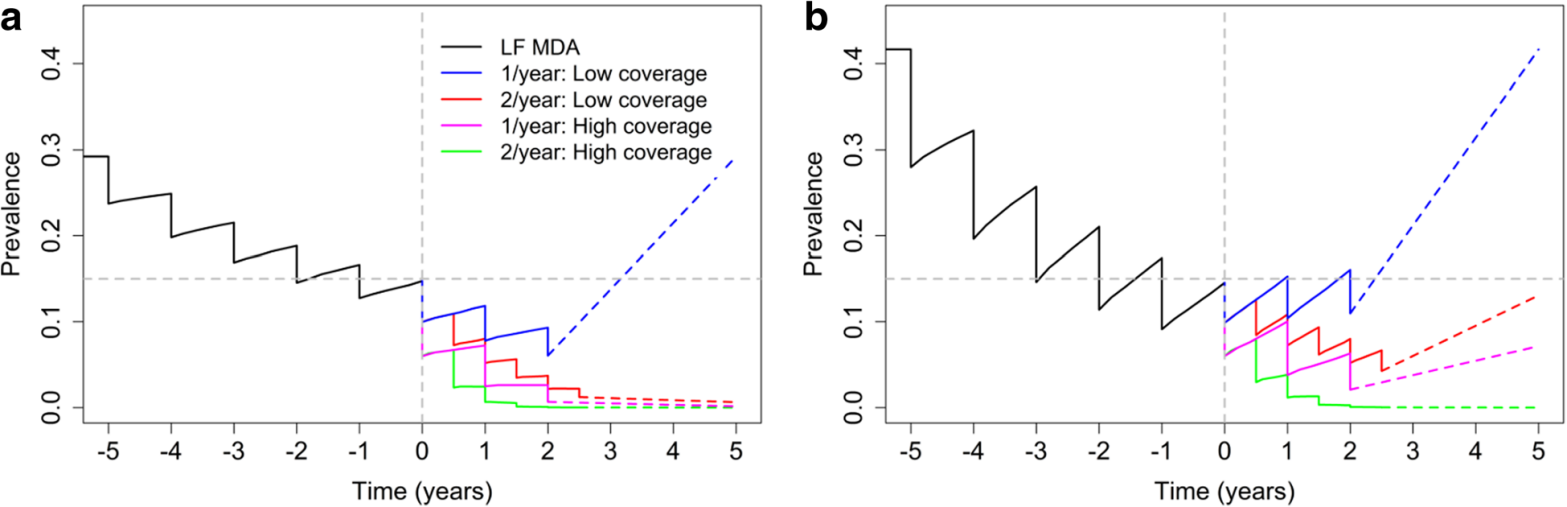
The effects of four different treatment interventions after five annual LF rounds with two different LF coverages (*black*). **a** The results with a LF coverage of 0.4, 0.4, 0.3 for pre-SAC, SAC and adults with an R_0_ of 1.67. **b** The results with a LF coverage of 0.7, 0.7 and 0.6 for pre-SAC, SAC and adults and assumes an R_0_ of 2.47. The* grey horizontal line* indicates a prevalence of 0.15 and the *grey vertical line* indicates the first round of the STH control treatment. The* dashed colored lines* represent the prevalence projections after MDA cessation

The success of different STH treatment regimens (coverage and frequency) is highly dependent on the baseline prevalence and transmission intensity as measured by R_0_. In Fig. 6, the effects of transmission intensity are shown for four different treatment regimens. We assume two different coverages during the 5 years of LF treatment, as defined for Fig. 4. These are classified as low LF MDA coverage (0.4, 0.4 and 0.3 for pre-SAC, SAC and adults, respectively) and high LF MDA coverage (0.7, 0.7 and 0.6 for pre-SAC, SAC and adults, respectively). Given the prior LF history and the baseline prevalence, we estimate the R_0_ to be 1.67 for low LF MDA coverage (Fig. 6a) and 2.47 for high LF MDA coverage (Fig. 6b). After five rounds of treatment, this results in a prevalence of 15% in both the low LF MDA coverage and high LF MDA coverage scenarios. We then analyse four different treatment regimens for the follow-up STH treatment (three years), where two different treatment frequencies (once and twice a year) and two coverage levels (defined as a proportion effectively treated) are examined. The successes of a low STH coverage of 0.5 and high STH coverage of 0.8 for pre-SAC and SAC (adults are assumed to have a 10% lower coverage level) are investigated.

Even though the baseline prevalence in both cases is 15% (Fig. 6), the success of the treatment regimens varies greatly in the two scenarios. With the low LF MDA coverage scenario (Fig. 6a), three out of the four regimens are successful in eliminating transmission, whilst only the regimen with annual low STH MDA coverage is not successful in interrupting hookworm transmission within three years. For the high LF MDA coverage scenario (Fig. 6b), although the baseline prevalence is identical, it proves to be more difficult to eliminate hookworm. Only the most intense regimen, bi-annual high STH coverage, successfully eliminates hookworm. With the other regimens, the infection is predicted to bounce back to its intrinsic endemic steady-state after STH MDA treatment ceases assuming there is no further treatment in subsequent years.

**Fig. 7:**
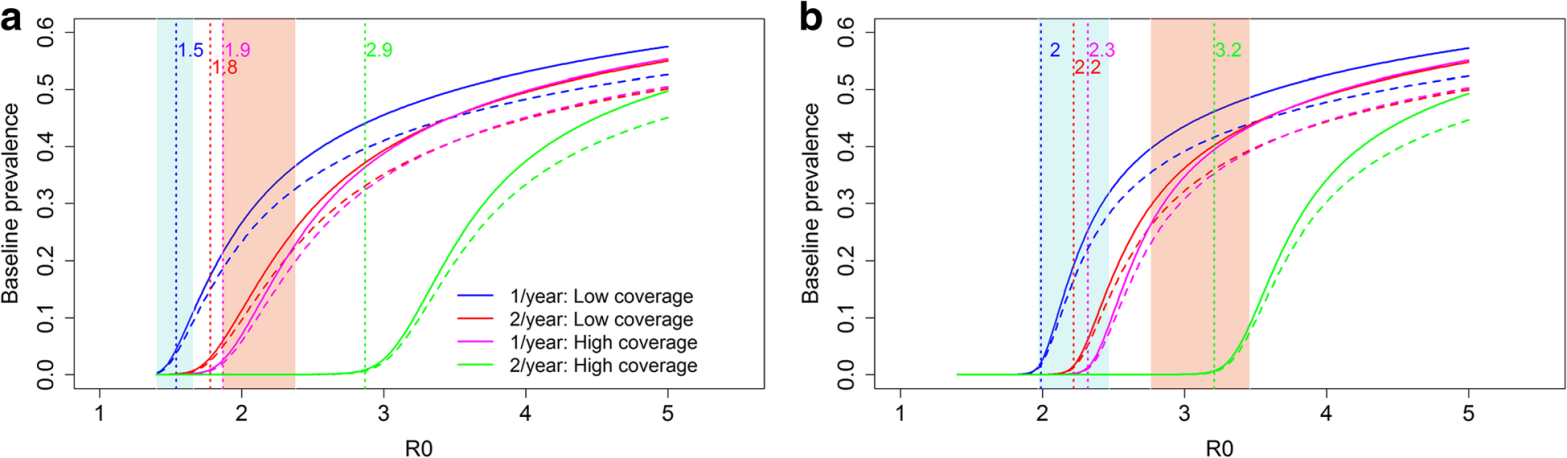
The impact of different treatments for the hookworm elimination programme. **a** The results with a LF coverage of 0.4, 0.4 and 0.3 for pre-SAC, SAC and adults respectively. **b** The results with a LF coverage of 0.7, 0.7 and 0.6 for pre-SAC, SAC and adults respectively. The shaded area corresponds with the expected R_0_ based on the LF rounds at two different prevalence regions: low (*blue*, 5–15%) and high (*red*, 20–30%). The *dotted vertical lines* indicate the predicted (deterministic) R_0_ values at which elimination occurs. The *curved lines* represent the prevalence of hookworm at baseline. The *solid lines* represent the true prevalence and the dashed lines the measured prevalence

Figure 7 records the predicted results of the STH programme, three years of MDA with a frequency of once or twice a year (identical conditions to those presented in Fig. 6). The shaded area corresponds to the estimated R_0_ based on the five LF MDA rounds (Fig. 4) assuming a low LF coverage (Fig. 7a) and a high LF coverage (Fig. 7b). The dotted vertical lines indicate the (deterministic) R_0_ values at which interruption of transmission is predicted to occur with the defined level of coverage. For an intervention to be successful, the R_0_ values at which elimination is predicted to occur, need to be larger than the estimated R_0_ value in the defined setting/community (Fig. 7).

For example, if the low LF MDA coverage regimen is considered, the R_0_ is expected to fall within 1.37–1.67 when the baseline prevalence is between 5–15% (Fig. 4a, Fig. 7a). Implementing the lightest treatment programme (treating once a year with a low coverage) may not be sufficient to achieve elimination. The reason is that the R_0_ value at which interruption of transmission is predicted to occur falls within the expected R_0_ range (the blue shaded area) when a low LF coverage is achieved and the baseline prevalence is between 5–15%. This is in contrast with the most intense strategy (treating twice a year with a high coverage), this programme is expected to eliminate transmission settings with an R_0_ < 2.9. This is sufficient for both prevalence ranges in the low LF MDA coverage settings (Fig. 7a). The two remaining control strategies (treating twice a year with a low coverage and treating once a year with a high coverage) are both predicted to achieve elimination in the low LF MDA coverage setting with a baseline prevalence between 5–15%, but are unlikely to be effective in eliminating hookworm in communities with the high baseline prevalence range (20–30%).

For the high LF MDA coverage scenario (LF MDA coverage of 0.7, 0.7 and 0.6 for pre-SAC, SAC and adults, respectively) the situation is different (Fig. 7b) as the transmission intensities are higher and therefore it is more difficult to achieve interruption of transmission. Only the most intense intervention is likely to achieve elimination in the 5–15% prevalence settings with an R_0_ up to 3.2 (Fig. 4b). The R_0_ values, at which interruption of transmission is predicted to occur, of the remaining three regimes fall within the R_0_ range that is predicted for the 5–15% prevalence range and are therefore likely to fail to achieve elimination after STH MDA cessation. When the prevalence falls within 20–30%, the R_0_ is expected to be between 2.76 to 3.36, and even the most intense regime may not be sufficient in this case. The most intense strategy is likely to eliminate hookworm in settings with R_0_ < 2.9. Therefore, further treatment is likely to be required in an area with a very high transmission intensity as defined by an R_0_ value of 3.0 and above.

## Discussion

This research highlights the benefits arising from the integration of different strands of NTD control under a common analysis and monitoring and evaluation umbrella. Understanding what may happen to both the prevalence and intensity of STH infection following a successful LF treatment programme, requires information from the LF programme operators on the level of MDA coverage achieved (and precisely who is treated at each round, stratified by age grouping). Taking hookworm as an example, our analyses illustrate how STH transmission intensities, as measured by R_0_, can be estimated given two data sources. These data sources include the prevalence of infection at baseline (defined as when LF treatment ceases and just before STH targeted treatment intensifies) across all age groups, and age-stratified treatment coverage data throughout the LF MDA period. Knowing the magnitude of STH transmission intensity is essential for identifying the MDA coverage requirements needed to achieve interruption of transmission once the LF programme ends. Our results show that baseline STH prevalence alone, after the cessation of LF treatment, is insufficient with regards to informing policy due to a high dependence on the coverage levels achieved over time in the past LF control programme. WHO guidelines aim to provide advice to governmental bodies on MDA policies based on prevalence data [34], with more countries participating in community-wide MDA programmes to control STH, prevalence data alone will not be sufficient to better design control and elimination programmes.

The model parameters adopted in this paper were estimated from a study performed in India [32] and, since transmission dynamics differ between and within countries, the results of our study may not directly translate to other endemic regions. However, the methods used are more generally applicable and can be applied to other settings. Parameter estimation techniques using longitudinal and cross-sectional data can be performed to incorporate country-, region-, or village-specific transmission dynamics. To make accurate predictions on a country-by-country basis, details of any previous LF programmes, combined with baseline data from the community of interest, are a necessity to estimate country-specific transmission intensities and to help design STH follow-up programmes that aim towards STH elimination. To accurately assess longitudinal trends in STH infection, it is essential to monitor not just SAC but also pre-SAC and adults. In other words, monitoring and evaluation programmes must be fully cross-sectional by age.

The data collected during the study performed by Sarkar et al. [32] on infection levels in individuals were based on the use of the McMaster technique. The model fitting for the parameters was based on data collected during detailed epidemiological research studies [32] and hence are assumed to be of good quality. However, we expect that epidemiological data collected during national programmes may be of a lower quality due to the high volume of samples collected and requiring analysis. Moreover, if STH prevalence is moving towards elimination, the intensity of infection is likely to drop and diagnostics with a higher sensitivity, such as qPCR [33], may be needed to provide accurate information.

Community-wide MDA coverage is not typically provided during national STH control programmes [26, 29] at present. However, the treatment of adults is necessary when the interruption of transmission is the objective, especially for hookworm where this age-group suffers from both the highest worm burdens and highest prevalence of infection [11, 12, 25]. In areas with a history of good LF control, it is likely that the prevailing infrastructure and data collection procedures are able to provide information on yearly coverage achieved and perhaps even changes in STH prevalence during the LF programme. As the emphasis in some countries shifts from morbidity control to the elimination of STH transmission [5, 13], it is important to collect prevalence data in adults and to record MDA coverage at a community-wide level stratified by age and with longitudinal information on individuals [26].

Currently recorded MDA coverage data have many limitations, whether for LF, schistosomiasis or STH control, as noted by Shuford and colleagues [29]. For example, few studies have recorded age-stratified longitudinal data. Further, many country databases may contain discrepancies between the recorded coverage and the true coverage achieved [35]. This is because the population size and age structure, and hence the denominator in calculating the proportion treated, may be uncertain. Moreover, what is meant by coverage and compliance differs between published studies. In our analyses, we define coverage as the proportion of the population or age group actually taking the drug. We do not consider people who systematically non-comply. However, we do recognize the potential impact of systematic non-compliance on the likelihood of interrupting transmission, depending on the disease dynamics and proportion of the population systematically not participating in MDA programmes [30, 36]. Individuals may decide not to participate in an MDA programme because they are asymptomatic; they suffered from, or fear suffering from, potential side-effects; they forget to take the drugs; or due to another illnesses [29]. In the model calculations, treatment coverage is assumed to be at random at each round of MDA within an age group. Future implementation research needs a greater focus on detailed compliance patterns over time and the local demography associated with treatment uptake. Since longitudinal compliance data are very limited, and when collected there is a high degree of uncertainty in the denominator (who was treated and local demography), we decided not to include non-compliance in this study. However, a detailed description on how to include non-compliance information in transmission dynamic analyses of MDA impact is described in other studies [30, 36].

In this study, we assumed that villages were closed epidemiological units and that there was no contamination between villages through migration. However, such migration between villages would mean contamination of disease-free villages with infectious material is likely *via*, for example, open defaecation. Some areas are known to have a large number of migrant labourers returning back to their home village during various festivals at different times of the year [32]. Ensuring that these individuals are included in deworming programmes is likely to increase the chance of achieving transmission elimination. To date, the migration and movement of individuals between villages and the concomitant impact on re-infection has not been studied in detail in the context of helminth infection control. However, it is an area of obvious importance, especially if attempts at transmission elimination are to be successful.

## Conclusions

Recently, there has been a shift within STH research from morbidity control to interruption of transmission [5, 11–13]. Trials are ongoing to investigate whether interruption of STH transmission is possible with community-wide MDA alone when implemented after a successful LF programme [5, 13]. This study shows the impact of historic LF treatment on hookworm prevalence and highlights the importance of the transmission rate, R_0_, on the expected success of an elimination programme. Baseline prevalence data at baseline are often collected, however these data alone are not sufficient to estimate the elimination potential of a programme. Additional data on both coverage and duration of LF MDA programmes, at the community level are needed to estimate the underlying transmission intensity (R_0_) prior to LF MDA and thus accurately predict the potential for STH elimination in a given setting.

## Abbreviations

epg: eggs per gram
LF: Lymphatic filariasis
MDA: Mass Drug Administration
NTD: Neglected Tropical Diseases
Pre-SAC: Pre-school aged children
SAC: School-aged children
STH: Soil-transmitted helminths
WHO: World Health Organization
